# Functional, eco-friendly, and starch-based nanocarriers with sustained release of carvacrol for persistent control of tomato gray mold

**DOI:** 10.1007/s44297-023-00014-9

**Published:** 2023-11-29

**Authors:** Wenxuan Shang, Qiuyu Xiong, Zhengang Xie, Jingli Cheng, Bin Yu, Haonan Zhang, Yehua Su, Jinhao Zhao

**Affiliations:** 1 Key Laboratory of Biology of Crop Pathogens and Insects of Zhejiang Province, Ministry of Agriculture Key Lab of Molecular Biology of Crop Pathogens and Insects, Zhejiang University, Hangzhou, 310058 People’s Republic of China; 2 Bayinaobao Industry Park, Alxa Economic Development Zone, Alxa League, Inner Mongolia People’s Republic of China

**Keywords:** Carvacrol, Starch, Nanopesticide, Sustained release, *Botrytis cinerea*

## Abstract

Carvacrol (Car) is a promising botanical fungicide. However, the volatile and easily oxidizable characterization of Car hinders its practical application. Herein, an eco-friendly, starch-based nanopesticide (Car@ACS) was successfully constructed by encapsulating of Car within acetylated corn starch, enabling sustained release to prolong its efficacy. In vitro release experiments showed that the release of Car from commercial carvacrol soluble concentrate (Car SL) was 98.4% within 4 h, while that of Car@ACS released 36% within 4 h and 79% within 48 h, demonstrating the endowed sustained release property of the starch nanoparticles. Meanwhile, in vitro antimicrobial activity experiments revealed the fungicidal ability of the prepared Car@ACS and carvacrol technical (Car Tech) with the same concentration of active ingredient to be comparable *Botrytis cinerea.* In the 7-day pot experiment, the efficacy of the Car@ACS group (56.9%) against *B. cinerea* was significantly greater than that of the Car SL group (33.3%)*.* In addition, the Car@ACS formulation improved the foliar retention of Car compared to Car SL in hydrophilic cucumber and hydrophobic peanut leaves. Finally, biosafety tests indicated that Car@ACS did not significantly affect the germination or growth of tomato seedlings. Therefore, the advantages of long-lasting disease control properties, improved foliar retention and eco-friendliness indicate that Car@ACS has significant potential to facilitate the application of volatile botanical fungicides.

**Supplementary Information:**

The online version contains supplementary material available at 10.1007/s44297-023-00014-9.

## Introduction

Carvacrol (Car) is a phenolic monoterpenoid compound found prominently within essential oils such as oregano (*Origanum vulgare*), thyme (*Thymus vulgaris*), and wild bergamot (*Citrus aurantium bergamia*) [1]. Car has been acknowledged for its antimicrobial, antioxidant, anticancer, and anti-inflammatory characteristics in clinical contexts owing to its broad spectrum of biological activities [1, 2]. Hence, Car offers a diverse array of functional potential in the pharmaceutical [3], food [4, 5], cosmetic [6, 7], and agricultural [8, 9] domains. Furthermore, no cytotoxic effects of Car on human cells were observed, even at the highest tested concentration of 90 mg/mL. Therefore, Car is generally recognized as safe (GRAS) by the US Food and Drug Administration (FDA) and approved by the European Commission [10, 11]. Car exhibits antimicrobial activity superior to that of volatile compounds in essential oils, owing to its possession of free hydroxyl groups, hydrophobic nature, and phenolic constituents, and has been demonstrated to possess antifungal activity against a broad spectrum of plant pathogens [12, 13]. Compared with traditional chemical pesticides, Car, a volatile botanical fungicide, exhibits the following advantages: (i) low toxicity; (ii) rapid decomposition; (iii) biocompatibility; and (iv) low exposure with almost no emission issues [14]. However, Car is volatile, readily oxidizable and insoluble in water, resulting not only in its short duration of efficacy and increasing frequency of sprinkling in production, greatly reducing its utilization but also in the conventional formulation of organic solvents, which are often used in large quantities [15]. In light of the demand for green and sustainable agriculture, there is a need to develop a new water-based eco-friendly formulation that enhances the utilization of Car [16, 17].

Here, a promising approach was presented to encapsulate Car without cross-linking and to achieve the sustained release of Car by constructing nanocarriers. This method reduces the volatilization of Car by providing a sustained, slow release, thus increasing the effective utilization of Car. Simultaneously, this method performed better in terms of water dispersion and exposure risk compared to traditional formulations. Accordingly, various types of materials have been used to load volatile essential oils, such as gelatin-gum arabic [18], sodium alginate [19, 20], chitosan [21], isocyanates [22], silicon dioxide [23, 24], polystyrene [25], and metal–organic frameworks [10]. However, pesticide use in agricultural production leads to the retention of pesticide formulations in the environment [26]. Hence, for the purpose of sustainable agriculture, materials that pose potential risks to the environment, for example, some metal–organic frameworks containing heavy metal ions and substances that degrade slowly or do not degrade at all in the natural environment (e.g., inorganic nanoparticles and organic microplastics), are not suitable carriers of pesticides. Notably, some materials were made of naturally degradable macromolecules, such as gelatin, gum arabic, and sodium alginate. However, the pesticide microcapsules prepared from these materials tend to have large particle sizes (> 5 μm) and wide particle size distributions due to the nature of the materials and easily rupture and collapse [27]. This results in the explosive release of the encapsulated active ingredient and subsequent loss of sustained release [28]. Therefore, developing cost-effective, stable, biodegradable, and biocompatible nanocarriers for drug delivery is crucial.

Starch, a macromolecular carbohydrate stored in plants, is the second most abundant following cellulose in nature [29, 30]. For the past few years, starch and its modified derivatives have become highly promising vehicles for drug delivery [31, 32]. Especially in the field of pesticide delivery, starch offers the advantages of being inexpensive, renewable, sustainable, and biodegradable [33, 34]. The acetylation reaction required mild conditions, and the process can be fully completed at room temperature [35]. The introduced acetyl groups significantly enhanced the solubility of starch in organic solvents [36]. Hence, the modified starches could be easily converted into nanoparticles based on emulsification to deliver pesticide [37]. At the same time, nanoparticles were better dispersed in aqueous solutions and remained uniformly distributed when sprayed on crop surfaces [17, 38, 39]. However, when conventional pesticide droplets were applied to crops, the particles had a tendency to aggregate into large particles on the surface of the crops instead of being individually dispersed at the nanoscale, which reduced the effectiveness of pesticide [40]. Therefore, it is of important significance to develop a simpler, eco-friendly, size-stable, water-dispersible starch-based nanopesticide carrier that is an effective means to promote sustainable agriculture.

**Fig. 1:**
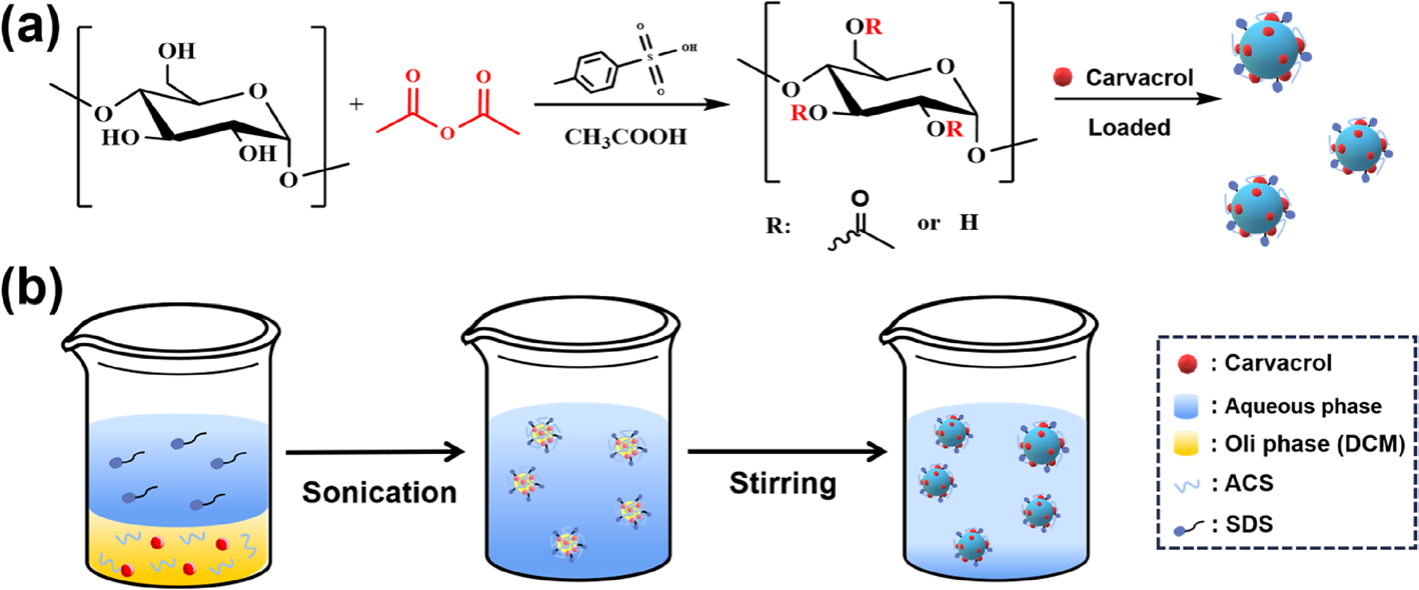
**a** Synthesis route of acetylated corn starch, **b** Schematic diagram of the synthesis of Car@ACS nanocarriers

In this study, a nanocarrier was developed using corn starch (CS) as a raw material. Acetylated corn starch (ACS) was obtained by esterifying CS with acetic anhydride, and the volatile substance Car was loaded by emulsification and the solvent evaporation method (Fig. 1a and b). Meanwhile, the characterizations of Car@ACS were conducted in detail to screen for the formulation with the best stability and investigate its sustained release performance, control efficiency against tomato gray mold (*Botrytis cinerea*), and foliar retention. The promise of ACS nanocarriers in pesticide delivery systems was demonstrated.

## Results

### Synthesis and characterization

Modification of corn starch containing many hydroxyl groups in the molecule with acetic anhydride to obtain acetylated corn starch with adjustable hydrophilicity (ACS). After the reaction, the hydroxyl group on the corn starch was replaced by an acetyl group. The extent to which the hydroxyl group on each glucose unit in the starch was replaced was represented by the DS. Since each glucose unit has three meridian groups that can be substituted for the glucose unit of starch, the maximum DS was 3. As shown in Table S1 and Fig. S1, the DS of ACS showed an increasing and later decreasing trend with the increase of the molar ratio of the amylose unit to acetic anhydride. This was because esterification was a reversible reaction, and by increasing the amount of reactants, the reaction equilibrium shifted in favor of the esterification reaction, thus favoring DS. However, because of the reversibility of the reaction and the degradation of starch by acids, the DS of the product did not reach the theoretical value, even when the molar ratio of glucosyl to anhydride was increased [41]. The DS of ACS reached a maximum of 2.88 when the reaction ratio reached 1:5.

**Fig. 2:**
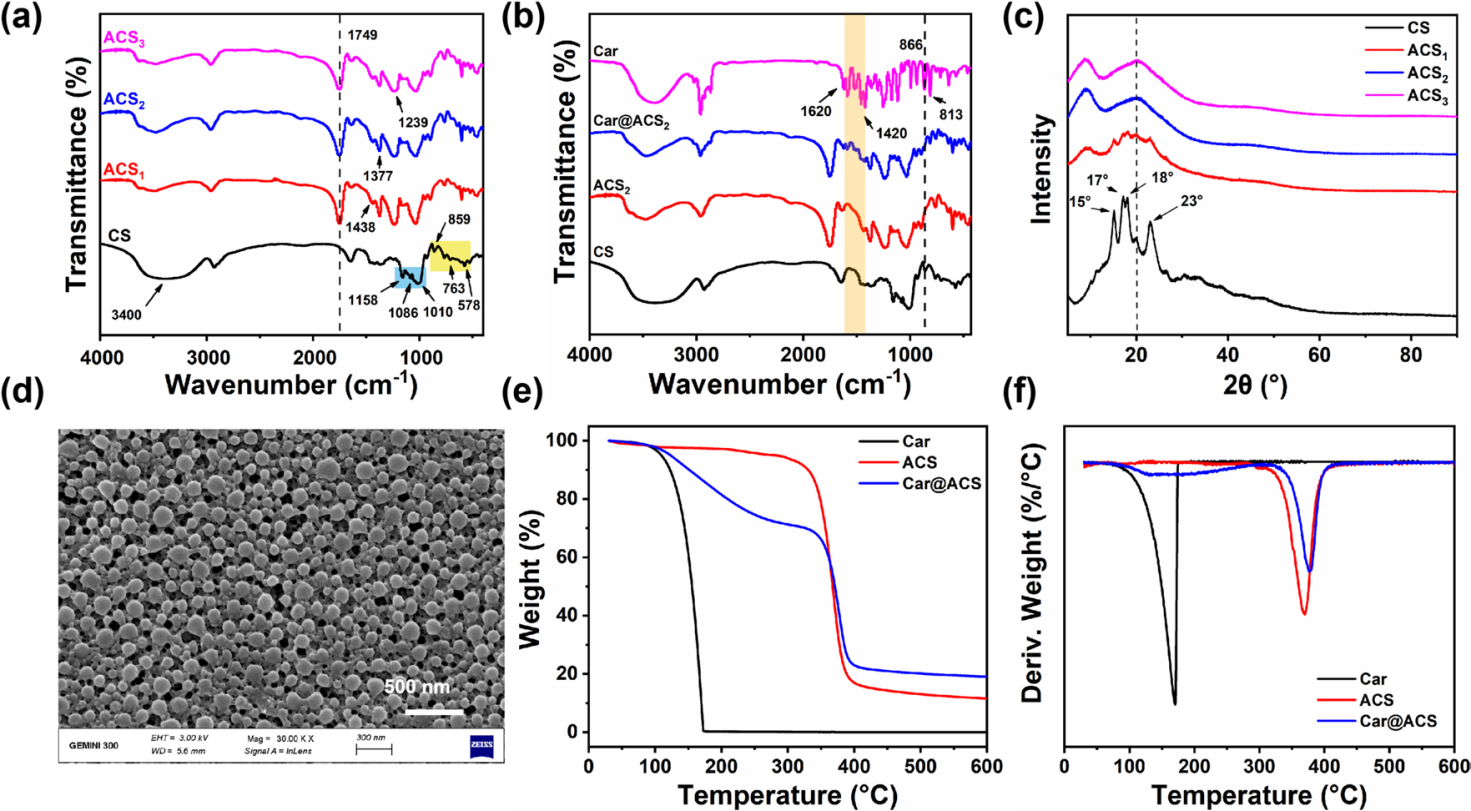
FTIR spectra of (**a**) different acetylated corn starch (ACS_1_, ACS_2_, and ACS_3_), CS and (**b**) ACS_2_, Car@ACS_2_, and Car. **c** XRD patterns of different ACS (ACS_1_, ACS_2_, and ACS_3_) and CS characterization spectra. **d** SEM image of the Car@ACS_2_ particles. **e** TGA, (**f**) DTG patterns of Car, ACS, and Car@ACS particles

The FT-IR spectra of CS and acetylated starch are shown in Fig. 2a. In the spectrum of CS, there were significant absorbances at 1158, 1086, and 1010 cm^−1^, which belonged to the stretching of the C–O bonds of the starch main chain [42]. Additional characteristic absorption peaks appeared at 859, 763, and 578 cm^−1 and^ were related to the stretching vibrations of the entire hydrogen-free glucose ring [43]. An extremely broad band caused by hydrogen-bonded hydroxyl groups appeared at 3400 cm^−1^. Compared with CS, the modified ACS showed some new absorption peaks at 1749, 1377, and 1239 cm^−1^ from the carbonyl C = O stretching vibration, CH_3_ symmetry deformation vibration, and carbonyl C–O stretching vibration, respectively [44]. These new bands confirmed that the esterification reaction occurred in CS. The intensities, areas, and heights of these peaks depend largely on the DS. With an increase in the DS, the intensities, areas, and heights of the peaks at approximately 3400 cm^−1^, which were related to the OH vibrations, gradually decreased, and these peaks almost disappeared when the DS reached 2.88, indicating that the hydroxyl group was completely involved in the reaction [42]. In contrast, the parameters including the peaks related to C = O at 1749 cm^−1^ and 1438 cm^−1^ and those related to acetylmethyl at 1239 cm^−1^ and 1377 cm^−1^ increased significantly as the DS increased [45]. For the spectra of Car@ACS, the bands between 1620 and 1420 cm^−1^ could be assigned to C–C stretching of the aromatic ring of the carvacrol molecule, and the C = C stretching of the aromatic ring was found at 866 and 813 cm^−1^ (Fig. 2b) [46].

The X-ray diffractograms of CS and ACS showed differences between the DSs (Fig. 2c). The CS particles illustrated a typical grain diffraction pattern with four high-intensity peaks at 15°, 17°, 18° and 23° [47]. However, the ACS particles showed a gradual disappearance of the peaks at 15°, 17°, 18° and 23°, which was related to the crystallinity reduction after esterification [48]. The introduction of acetyl groups attenuates the formation of inter- and intramolecular hydrogen bonds, thereby disrupting the original double-helical structure [49]. ACS showed a new peak at 9°, appearing as a diffusion peak of acetylated starch. The peak at 21° became more pronounced as the DS increased. This pattern is characteristic of V-type single-helix structures [50]. Therefore, the crystallinity of ACS became V-shaped, and the molecular structure changed from a double-helix mode to a single-helix mode. X-ray diffraction results showed that during the esterification process, the crystalline structure of natural starch was disrupted, and a new structure of acetylated starch was formed.

Figure 2e, f shows the weight change with increasing temperature in the N_2_ atmosphere for several samples. The weight loss of Car@ACS nanoparticles was divided into two stages: in the first stage, when the temperature increased from 90 °C to 305 °C, the loaded Car as well as the active groups in the acetylated starch were degraded in the high-temperature environment; in the second stage, the temperature increased from 305 °C to 405 °C, primarily attributable to the decomposition of the acetylated starch molecules [51]. The weight loss curves of Car and ACS were also consistent with the speculation of the weight loss curve of Car@ACS.

**Fig. 3:**
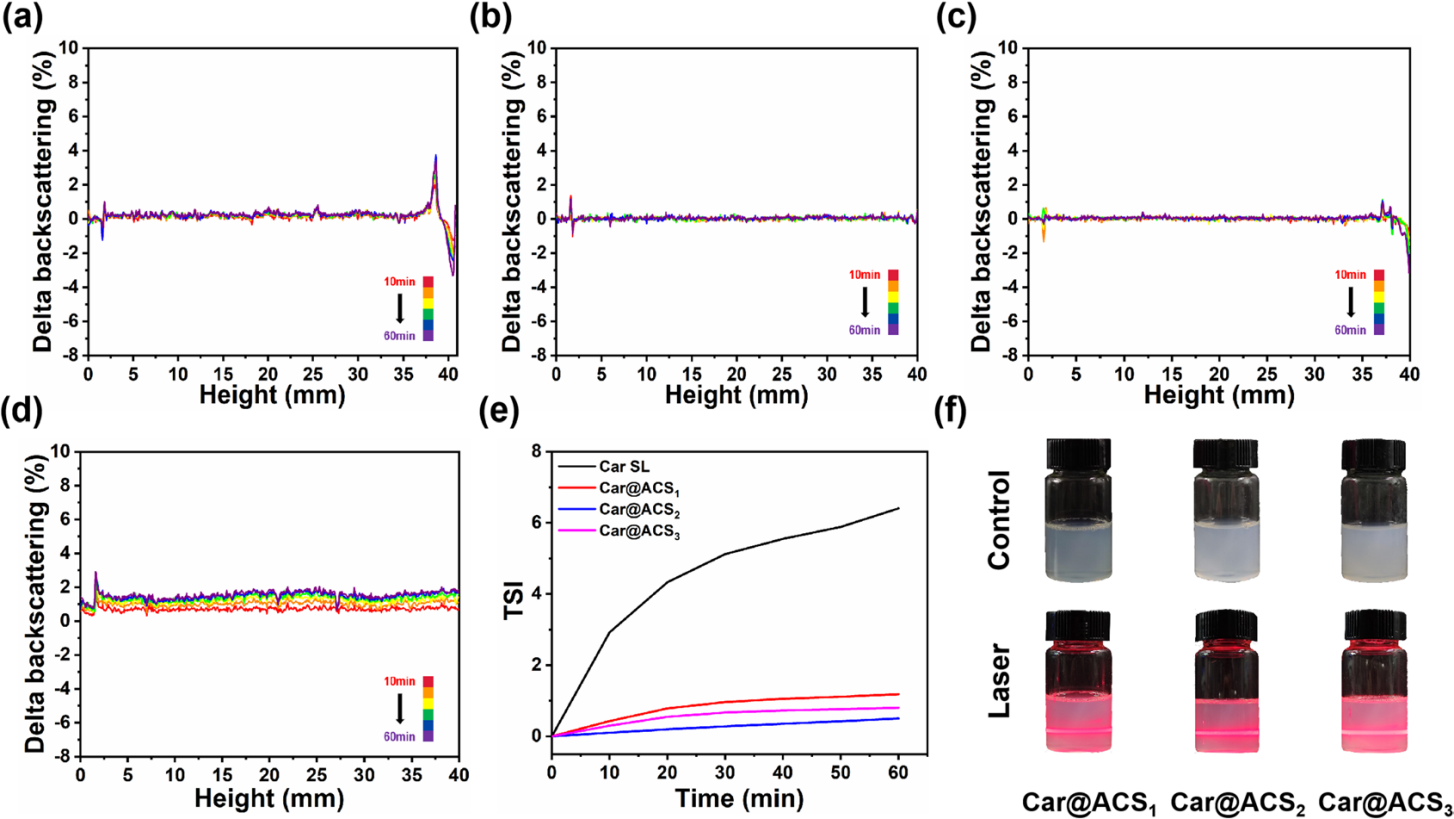
Backscattering profiles of (**a**) Car@ACS_1_, (**b**) Car@ACS_2_, (**c**) Car@ACS_3_, and (**d**) Car SL. TSI values of (**e**) different Car@ACS nanoparticles and Car SL. **f** Images of different Car@ACS formulations with the same Car concentration under laser radiation

The appearance of different Car@ACS formulations containing the same Car concentration (Fig. 3f) demonstrated that the appearances of the samples were not significantly different, and all samples formed a clear light path under laser irradiation. The morphology of the Car@ACS nanoparticles was further observed using SEM. As shown in Fig. 2d and Fig. S2., the average sizes of Car@ACS_1_, Car@ACS_2_, and Car@ACS_3_ were approximately 160.56 ± 2.8, 126.21 ± 1.3, and 128.27 ± 1.9 nm, respectively, as measured via DLS. The particle size distribution was uniform, and there was no obvious adhesive phenomenon, which suggested that the nanoparticles had good dispersion in water.

### Formulation stability analysis

Turbiscan analysis provided information on the migration of nanoparticles during formulation storage. Figure 3a–d shows the correlation curves between the scattered (%) and sample height (0–40 mm) and time (0–1 h) for the Car@ACS formulations and Car SL containing the same Car concentration. The scattered distributions of Car@ACS_2_ were slightly smaller than those of Car@ACS_3_, but they were all within ± 2%, indicating that no aggregation or migration occurred [52], which proved that they had a high stability of the formulation and were more stable than Car SL in the short term. In contrast, the Car@ACS_1_ formulation revealed a wide range of variations in the top-backwards scattering curve, suggesting that the particles had settled. In contrast, the commercialized Car SL underwent migration at any height, and the backscatter profile at the bottom was outside the ± 2% interval. TSI has been used to determine formulation stability: the lower the TSI value is, the higher the stability of the formulated samples [17]. In this study, all the TSI values of Car@ACS were lower than those of the commercialized Car SL (Fig. 3e). Meanwhile, the zeta potential indicated that the prepared nanoparticles had a higher negative charge than Car SL (Fig. S3). Finally, these results and those of SEM demonstrated that the well-stabilized preparation of Car@ACS formulations had small particles and highly negatively charged surfaces, resulting in less aggregation between particles. Based on the complexity, cost and stability of the preparation, Car@ACS_2_ was used for other subsequent performance evaluations. (Noted as Car@ACS).

### Release behavior and mechanism investigation

**Fig. 4:**
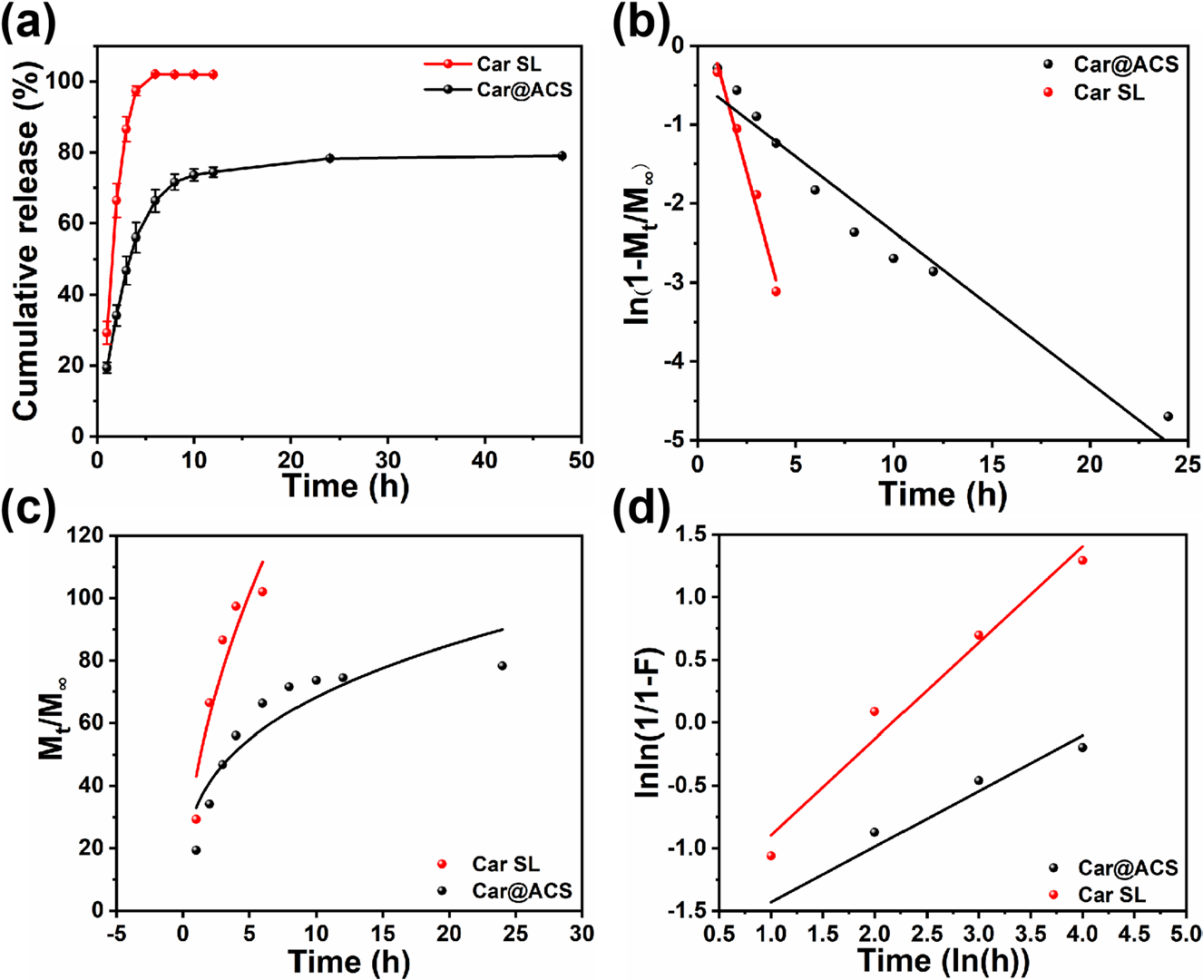
(**a**) Cumulative release profile and fitting curves of the (**b**) first-order model, (**c**) Ritger-Peppas model, and (**d**) Weibull model for the release of Car from Car@ACS and Car SL

The sustained release properties of fungicides are an effective method to increase their utilization in agricultural production. The cumulative release behavior of Car@ACS and Car SL formulations in an aqueous solution containing 20% ethanol (Fig. 4a). Car SL showed a rapid release at the beginning of the release period. The cumulative release rate of Car SL reached 98.4% after the 4 hand was not characterized by a sustained release. Whereas only 56.1% of Car was released from Car@ACS after 4 h, it was significantly slower than that from Car SL, indicating that the encapsulation prolonged the release time of Car. After 48 h, the cumulative release rate of Car@ACS was 78.8%.

To further explore the release of Car@ACS and Car SL, some classical models were fitted, such as the zero-order model, the first-order model, the Higuchi model, the Weibull model, and the Ritger-Peppas model (Supporting Information) [53, 54]. The release of Car from Car SL was more consistent with a first-order model (R^2^ = 0.995), indicating that the release of Car was dependent only on the remaining concentration remaining in the formulation. The cumulative Car release data from the Car@ACS nanoparticles showed the best fit to the Ritger-Peppas model (R^2^ = 0.926, *n* = 0.43), suggesting that the release of Car from the Car@ACS was consistent with Fickian diffusion (Fig. 4b–d, Fig. S5 and Table S2). Therefore, the ACS carrier delayed the release of Car better than the commercial formulation. Evidence has shown that the dissolution and diffusion behaviors of Car during particle degradation under the present experimental conditions jointly control the entire drug release process.

### In vitro fungicidal activity

**Fig. 5:**
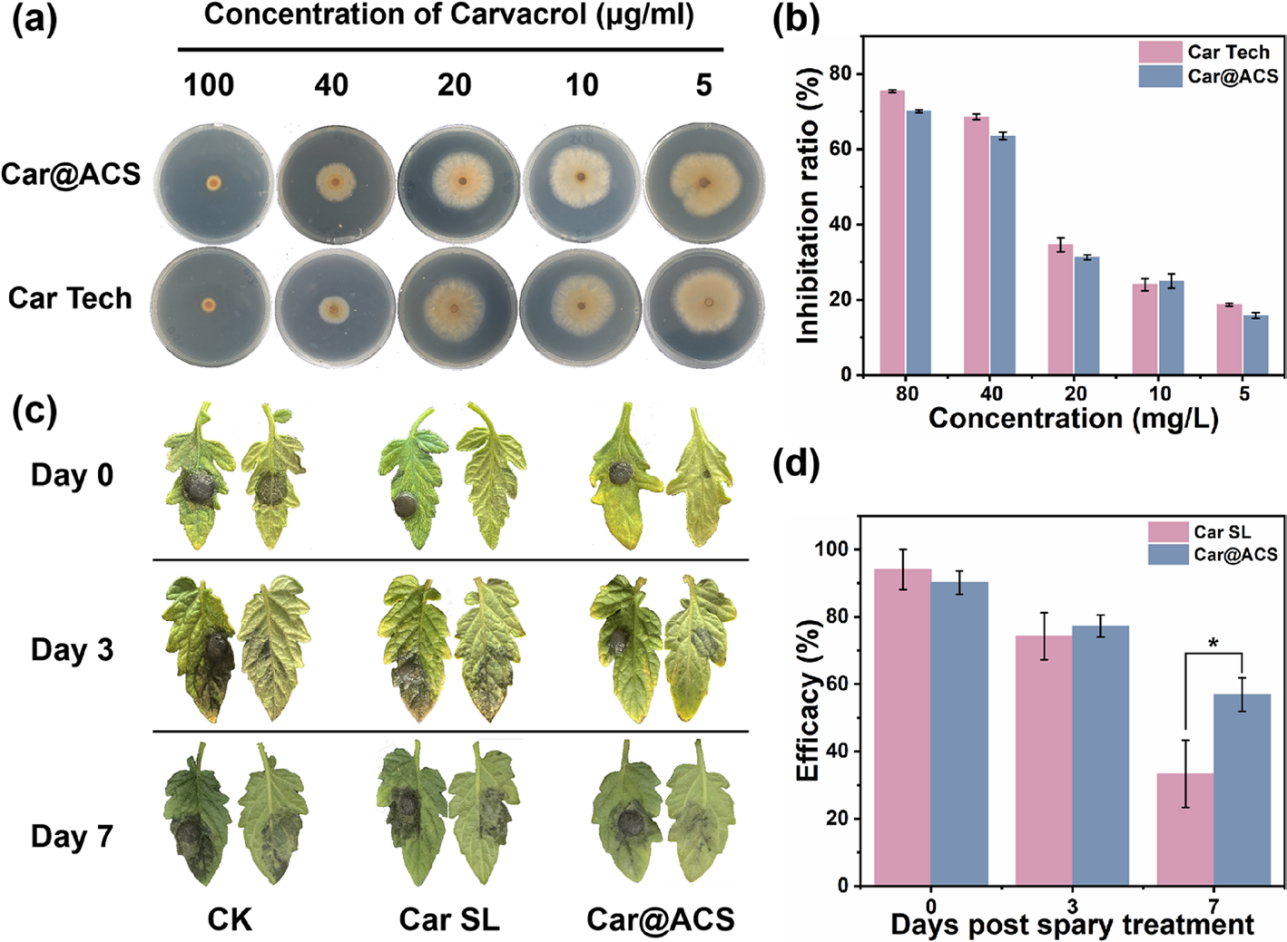
**a**, **b** In vitro antimicrobial activities of Car@ACS and Car Tech against *Botrytis cinerea.* **c**, **d**) Control efficacy of Car SL and Car@ACS against *Botrytis cinerea* on tomato leaves

*Botrytis cinerea* is a commonly observed fungus on tomato. *The *in vitro antimicrobial activity of Car@ACS was tested using Car Tech as a control Fig. S6. As shown in Fig. 5a,b, the antimicrobial activity was positively related to the Car concentration. Car Tech and Car@ACS containing the same Car concentration showed antimicrobial activity similar to that against *B. cinerea*. Meanwhile, the EC_50_ values of Car Tech and Car@ACS were 26.35 μg/mL and 32.22 μg/mL, respectively, which were not significantly different and were calculated based on the inhibition rates of different concentrations (Table S3). These results indicated that Car@ACS still had comparable antimicrobial activities to Car Tech.

### Control efficacy in pot experiment

To further investigate the control efficacy of Car@ACS nanoparticles against *B. cinerea*, a pot experiment was performed on tomato leaves (Fig. 5c, d). The Car@ACS and Car SL groups effectively inhibited fungal growth in the 0 d experimental group immediately after spraying. For the 3 d treatment, the control efficacy of the Car SL group was 74.2 ± 7%, slightly lower than that of the Car@ACS group (77.3%). However, when the fungus was inoculated 7 d after spraying, the inhibition rate of the Car SL group decreased to 33.3 ± 10%, and the inhibition rate of the Car@ACS group remained at 56.9 ± 5%, which was significantly better than that of the Car SL group. Car@ACS effectively prolonged the duration of commercialized Car formulations owing to the protection provided by the ACS carrier. In contrast, Car SL was much more unstable than Car@ACS, probably because the unprotected Car is unstable in response to light, temperature, microorganisms, and enzymes, which is not conducive to its persistence in the environment.

### Foliar retention and distribution

The retention and distribution of pesticides on the surface of crop leaves play a decisive role in their effective utilization. The important property of adhesion of the pesticide formulation droplets to leaves was evaluated by calculating the foliar retention before and after flushing.

**Fig. 6:**
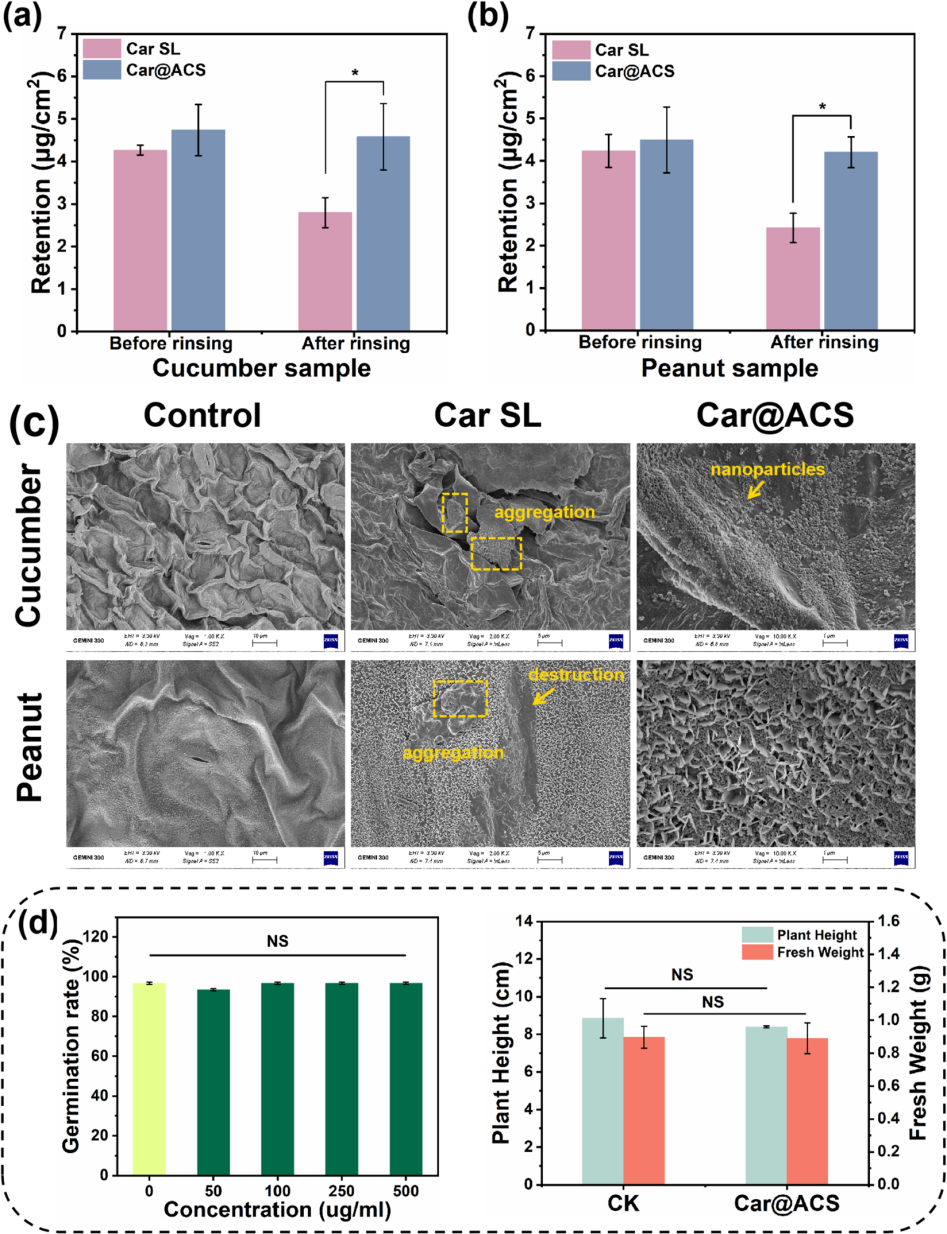
Retention of Car SL and Car@ACS on the surface of (**a**) cucumber and (**b**) peanut leaves before and after rinsing with water. (**c**) SEM images of cucumber and peanut leaf surfaces treated with the same Car concentration of Car SL and Car@ACS. **d** Biosafety assessment of blank Car@ACS nanoparticles on tomatoes, including seed germination rate, seedling height, and fresh weight

Before and after rinsing, the Car retention of Car@ACS on cucumber leaves was 1.11 and 1.64 times higher than Car SL, respectively (Fig. 6a). In addition, Car@ACS had 1.06 and 1.74 times higher Car retention on peanut leaves than Car SL (Fig. 6b). In addition, the Car retention rate of Car@ACS was higher than that of Car SL (Fig. S7) after spraying cucumber and peanut leaves, indicating that Car@ACS had a better rising resistance on the leaf surface. SEM revealed the cause of this phenomenon (Fig. 6c). The Car@ACS nanoparticles were uniformly distributed on the surface of the leaves. In addition, probably because of the high levels of emulsifiers in commercialized Car SL, a disrupted waxy layer was observed on peanut leaves, which significantly increased the risk of the crop being infected with the pathogen [17]. Notably, Car@ACS nanoparticles were embedded between the interstices of the waxy structure on the peanut leaf surface, which explains why Car@ACS had a better scouring resistance on the peanut leaves. Consequently, the car retention of Car@ACS is higher than that of Car SL due to the uniform distribution and embedding of nanoparticles on the blade surface.

### Biosafety investigation of Car@ACS

The results showed that Car@ACS nanoparticles did not significantly affect germination (Fig. 6d). Tomato seedlings were treated with 500 mg/L Car@ACS at the root level, and the fresh weight and root length of the tomatoes were measured after 30 d. There was no significant difference between the treatment results and the blank control. In summary, the Car@ACS nanoparticles exhibited good biocompatibility and biosafety for tomato seeds.

## Conclusion

In summary, a Car@ACS nanopesticide formulation with sustained release was successfully constructed by combining the volatile botanical fungicide car with ACS. During the process, the formulation was able to sustain the release of Car, showed no significant difference in fungicidal activity from Car Tech at a comparable concentration in vitro assay, and was significantly better than the commercialized Car SL in the 7 d pot experiment. This formulation prolonged its efficacy through sustained release, which reduced the frequency of the fungicide and enhanced the utilization rate of Car. Moreover, the formulation exhibited better stability and foliar retention than the commercial Car formulation. Biosafety experiments showed that Car@ACS based on starch material did not affect the germination rate or growth of tomato seedlings, suggesting that acetylated cornstarch could be used as a fungicide delivery vehicle. Overall, this study provides an approach for the preparation of nanopesticides that can prolong the duration and improve the utilization of the botanical fungicide Car, providing a novel approach to sustainable agriculture.

## Materials and methods

### Materials and chemicals

Food grade CS was purchased from Shanghai Fengwei Industrial Co. Ltd. (China). Car (96%) was purchased from Shanghai Macklin Biochemical Co., Ltd. (China). Sodium dodecylsulfate (SDS, 93%) was obtained from Shandong Yousuo Chemical Technology Co., Ltd. (China). *p*-Toluenesulfonic acid (99%) was purchased from Shanghai Rhawn Chemical Technology Co., Ltd. (China). Carvacrol soluble concentrate (Car SL, 5%) was purchased from Shanxi Dewei Biotechnology Co., Ltd. (China).

### Syntheses of acetylated corn starch

Corn starch (1.00 g, 6 mmol anhydroglucose unit) and acetic acid (5.0 mL) were placed in a three-necked flask equipped with a bulbous condenser tube. After the mixture was stirred for 15 min, the reaction temperature was raised to 70 °C, and 0.30 g of *p*-toluenesulfonic acid was added to the reaction flask and stirred for 15 min. Next, acetic anhydride in a 1:3, 5, or 7 to anhydroglucose units was added dropwise to the mixture and stirred for 3 h, and the reaction mixture was poured into distilled water to precipitate the product. The products were washed with distilled water and dried under vacuum at 40 °C for 24 h to obtain the degrees of substitution of acetylated starch: ACS_1_, ACS_2_, and ACS_3_.

### Preparation of the Car@ACS suspension

The Car@ACS nanoparticle suspension was prepared using the emulsification and solvent evaporation method. In brief, ACS (100 mg) and Car (50 mg) were dissolved in 1.0 mL of dichloromethane. Next, the solution was added to a reaction vial containing 0.2% wt SDS in water (10 mL). The suspension was pre-emulsified with a homogenizing emulsifier for 1 min (15,000 rpm) to obtain a milky white emulsion, followed by ultrasonication for 1 min (70% amplitude, 1 s on and then 2 s off). The obtained mixture was stirred overnight at room temperature (1,000 rpm) to obtain a suspension of Car@ACS nanoparticles.

### Characterization

The morphologies of the samples were observed using SEM (Zeiss, G300, Germany). ^1^H nuclear magnetic resonance (1H-NMR) spectroscopy was performed using a 300 MHz NMR spectrometer (Bruker 300 Ultrashield). The chemical compositions of the samples were measured using a Fourier transform infrared spectrometer (FT-IR, Nicolet Avatar370, Thermo Fisher, USA). X-ray diffraction (XRD, D8 Focus, Bruker, Germany) and thermogravimetric analysis (TGA, TGA2, Mettler Toledo, Switzerland) were performed. The zeta potential and size distribution of the samples were determined using a Mastersizer ZS-90 laser diffraction particle size analyzer (Malvern, U.K.).

### Determination of the degree of substitution

The degree of substitution (DS) of ACS was calculated according to ^1^H-NMR [55, 56]. ACS (10 mg) was accurately weighed in an NMR tube, and 0.6 mL of deuterated DMSO-*d6* was added. The acetylated starch was then completely solvated by sonication for 1 min, and then the solution composition was determined via ^1^H-NMR. Typically, the backbone signal of starch is in the range of 3.9–5.5 ppm. The strongest signal peak was for the proton on the acetyl group, with the proton peak of the methyl group of the acetyl group at 3.9–5.5 ppm.

The DS was calculated using the ratio of the signal peak area of the proton of the methyl group in the acetyl group to that of the proton of the glucose unit, which had seven protons (excluding the proton of the light group), using the following formula:

$$\mathrm{DS} = \frac{{7}{\mathrm{A}}_{\mathrm{ace}}}{{3}{\mathrm{A}}_{\mathrm{aqu}}}$$

where A_ace_ is the methyl signaling peak area, 1.9–2.1 ppm; and A_aqu_ is the signaling peak area of the amylopectin skeleton, at 3.9–5.5 ppm.

### Formulation stability analysis

The Turbiscan Lab Expert (Formulation, France) was used to analyze the stability of different Car@ACS and commercial carvacrol soluble concentrate (Car SL) [17, 57]. The formulation stability for Car@ACS and Car SL was performed as a variation in backscattering (ΔBS) profiles. Measurements were performed using an a2 near-infrared LED at a wavelength of 880 nm for 1 h. Experimental data were correlated in percentage with the light flux of two reference standards, consisting of a polystyrene latex suspension (absence of transmission and maximum backscattering) and silicon oil (maximum transmission and absence of backscattering). The Turbiscan Stability Index (TSI) can also be used to determine formulation stability [18]. The formula was as follows:

$$\mathrm{TSI}=\sum_\mathrm{i}\frac{\sum_h\mathrm{|}{\mathrm{scan}}_\mathrm{i}\left(h\right)-{\mathrm{scan}}_\mathrm{i-1}\left(h\right)\mathrm{|}}H$$

where *scan*_*i*_*(h)* is the light intensity of the *i*-th scan at a height of *h*, and *H* is the total height of the measured sample.

### Pesticide release behavior

The release performance of Car@ACS was investigated using the dialysis bag method [58]. Approximately 2 mL of Car@ACS and Car SL containing the same concentration of Car in a dialysis bag (molecular weight cut-off of 1 kDa) were placed in an aqueous solution containing 98 mL of 20% ethanol with uniform shaking at 25 °C. At different time intervals (0, 1, 2, 4, 8, 16, 24, and 48 h), 1 mL of the sample was removed and immediately replaced with 1 mL release medium. The Car content was assayed via HPLC (Shimadzu, LC-20A, Japan) using a reverse-phase chromatographic column and a 273 nm UV detector. The mobile phase consisted of acetonitrile and water (70:30, v/v) with a flow rate of 1.0 mL/min Fig. S4. The cumulative percentage release of Car was calculated using the following equation:

$$\mathrm{Cumulative}\;\mathrm{release}\;\left(\%\right)=\sum_{t=0}^t\frac{M_t}{M_0}\times100\%$$

where* M*_*t*_ is the amount of Car released at each sampling time point, t is the time of release of the Car-loaded nanoparticles, and *M*_*0*_ is the initial weight of the Car-loaded ACS nanoparticles.

### In vitro bioactivity assay

The antimicrobial activities of Car@ACS and Car Tech against *Botrytis cinerea* were investigated using the mycelial growth rate assay. *B. cinerea* was inoculated onto potato dextrose agar (PDA) media with a different concentration gradient, where the concentration of Car was 5, 10, 20, 40, and 100 μg/mL. The PDA medium was incubated in a constant temperature biochemical incubator (RXZ-31 °C) at 25 °C. Colony diameter was measured after 5 d using the crisscross method. Three parallel experiments were performed for each treatment group.

### Pot experiment

Approximately 2-month-old tomatoes were divided into three treatment groups at 0, 3, and 7 d. Tomato leaves from each group were uniformly sprayed with the same concentrations of Car@ACS and Car SL using a hand-held sprayer, and tomatoes sprayed with the same concentrations of ACS carrier and water as Car@ACS were used as controls. Then, on days 0, 3 and 7 after spraying, *B. cinerea* was inoculated onto tomato leaves. The three groups of samples were placed in a thermostatic light incubator for incubation (25°C, 75% humidity, light:dark, 12 h/12 h). After 36 h of incubation with *B. cinerea*, the diameter of the lesion was measured in two vertical directions. Each experiment was repeated five times. The disease control efficacy was calculated from lesion diameters.

### Retention and distribution

Car@ACS and Car SL were diluted to 100 μg/mL with deionized water, and 1.0 mL of the dilutions was sprayed onto hydrophilic cucumber leaves and hydrophobic peanut foliar surfaces. The leaves were then transferred to an inclined plane with a 45° inclination angle, dried naturally, sprayed with 10.0 mL deionized water to simulate the effect of rainfall, and dried naturally. Next, leaf samples of the same area before and after washout were collected with a punch for HPLC to determine the amount of Car on the leaf surface before and after rising. Finally, leaf samples of the same area before and after washout were collected using a puncher for HPLC to test the amount of Car on the leaf surface before and after the simulated rainfall washout. SEM was also used to observe the leaf surface before and after rainfall washout. Each experiment was repeated three times.

### Biosafety of Car@ACS toward tomato plants

Tomato seeds (cultivar “Zheza 809”) were treated with 5 mL of different concentrations of Car@ACS, which were diluted with deionized water to 0, 50, 100, 250, and 500 μg/mL, and then placed in a thermostatic light incubator for incubation (28 °C, 16 h/8 h day/night). After 5 days of vernalization, the germination rate of the seeds was calculated. Subsequently, seedlings with similar growth conditions from both the 0 and 500 μg/mL groups were transferred to pot culture. After 7 days of pot culture, the seedlings were treated with 10 mL of the same concentration of Car@ACS via root exposure. Finally, the physiological parameters of plant height and fresh weight were recorded on the 30th day after treatment with Car@ACS.

### Data analysis

For multiple group comparisons, one-way analysis of variance followed by Duncan’s test was performed using Statistical Product and Service Solutions. Different letters or asterisks (*) indicate significant differences at *p* < 0.05.

## Supplementary Information

**Additional file 1.** Supplementary Experimental Section. **Table S1.** The degree of substitution of different prepared corn starch ACS. **Fig. S1.** 1H-NMR spectra of CS, ACS_1_, ACS_2_, and ACS_3_.** Fig. S2.** SEM image, size distribution and DLS of different Car@ACS nanoparticles. Zeta potential of different Car@ACS nanoparticle and Car SL. **Fig. S3.** Turbiscan stability index (TSI) values and Zeta potential of Car@ACS_1_, Car@ACS_2_, Car@ACS_3_ and Car SL. A higher TSI value indicated a less stable formulation sample in different part. (Top: 40 – 30 cm, middle: 25 – 15 cm, and bottom: 10 – 0 cm). **Fig. S4.** The HPLC calibration curve for carvacrol. **Fig. S5. **Fitting curves of (a) zero-order model and (b) Higuchi model. **Table S2.** Release kinetics equations for Car SL and Car@ACS_2_. **Fig. S6.** Photographs of control (water) and ACS_5_ nanoparticles (20 μg/mL) against *Botrytis cinerea*. **Table S3.** The antimicrobial activities of Car Techand Car@ACS_2_ against *Botrytis cinerea*. **Fig. S7.** The Car retention rate of Car SL and Car@ACS_2_ after spraying onto cucumber and peanut leaves.

## Data Availability

The data that support the findings of this study are available upon reasonable request.
