# Supplementary material for: Functional, eco-friendly, and starch-based nanocarriers with sustained release of carvacrol for persistent control of tomato gray mold
Source: Crop Health. 2023 Nov 29;1(1):13. doi: 10.1007/s44297-023-00014-9 (PMC12825967; doi:10.1007/s44297-023-00014-9)
Supplement: Supplementary file 1 — Additional file 1. Supplementary Experimental Section. Table S1. The degree of substitution of different prepared corn starch ACS. Fig. S1. 1H-NMR spectra of CS, ACS1, ACS2, and ACS3. Fig. S2. SEM image, size distribution and DLS of different Car@ACS nanoparticles. Zeta potential of different Car@ACS nanoparticle and Car SL. Fig. S3. Turbiscan stability index (TSI) values and Zeta potential of Car@ACS1, Car@ACS2, Car@ACS3 and Car SL. A higher TSI value indicated a less stable formulation sample in different part. (Top: 40 – 30 cm, middle: 25 – 15 cm, and bottom: 10 – 0 cm). Fig. S4. The HPLC calibration curve for carvacrol. Fig. S5. Fitting curves of (a) zero-order model and (b) Higuchi model. Table S2. Release kinetics equations for Car SL and Car@ACS2. Fig. S6. Photographs of control (water) and ACS5 nanoparticles (20 μg/mL) against Botrytis cinerea. Table S3. The antimicrobial activities of Car Techand Car@ACS2 against Botrytis cinerea. Fig. S7. The Car retention rate of Car SL and Car@ACS2 after spraying onto cucumber and peanut leaves. [file 44297_2023_14_MOESM1_ESM.docx]

Appendix A. Supplementary data

**Functional,** **eco-friendly, and starch-based nanocarriers with sustained release of carvacrol for persistent control of tomato gray mold**

Wenxuan Shang^a^, Qiuyu Xiong^a^, Zhengang Xie^a^ , Jingli Cheng^a^, Bin Yu^a^, Haonan Zhang^a^, Yehua Su^b*^ & Jinhao Zhao^a*^

^a^ Key Laboratory of Biology of Crop Pathogens and Insects of Zhejiang Province, Ministry of Agriculture Key Lab of Molecular Biology of Crop Pathogens and Insects, Zhejiang University, Hangzhou 310058, P. R. China

^b^ Bayinaobao Industry Park, Alxa Economic Development Zone, Alxa League, Inner Mongolia, P.R. China

* Corresponding authors:

E-mail addresses: jinhaozhao@zju.edu.cn (J. Zhao), suyehua@maxunitech.com (Y. Su)

**Supplementary Experimental Section**

*Release kinetics investigation*

The release kinetics of Car from Car@ACS and Car SL were analyzed using zero-order, first-order, Higuchi, Ritger Peppas, and Weibull models as follows:

$$\text{Zero-order model: }\frac{\text{M}_{\text{t}}}{\text{M}_{\text{∞}}}\text{=kt (1)}$$

$$\text{First-order model: }\frac{\text{M}_{\text{t}}}{\text{M}_{\text{∞}}}\text{=1-}\text{e}^{\text{-kt}}\text{ }\text{ }\text{ (2)}$$

$$\text{Higuchi model: }\frac{\text{M}_{\text{t}}}{\text{M}_{\text{∞}}}\text{=k}\text{t}^{\text{1/2}}\text{ (3)}$$

$$\text{Ritger Peppas model: }\frac{\text{M}_{\text{t}}}{\text{M}_{\text{∞}}}\text{=k}\text{t}^{\text{n}}\text{ (4)}$$

$$\text{Weibull model: }\frac{\text{M}_{\text{t}}}{\text{M}_{\text{∞}}}\text{=1 -}\text{exp}^{\text{-at}^{\text{b}}}\text{ }\text{ }\text{ (5)}$$

$$\text{Weibull model: lnln(}\frac{\text{1}}{\text{1-F}}\text{) = blnt + lna }\text{ }\text{ (6)}$$

Where *M_t_* is the amount of fungicide released at time t, *M_∞_* is the maximal amount of the released fungicide at infinite time, *k* is the rate constant of the fungicide, n is the diffusion exponent (Fickian diffusion (*n* ≤ 0.43), non-Fickian (0.43＜*n*＜0.85), and Case-II transport (*n* ≥ 0.85) ); F and time are the cumulative release and time , a and b are constants.

**Table S1.** The degree of substitution of different prepared corn starch ACS

| **Code** | **Reaction rate** | **Degree of substitution (DS)** |
| --- | --- | --- |
| **ACS_1_** | 1:3 | 1.58 |
| **ACS_2_** | 1:5 | 2.88 |
| **ACS_3_** | 1:7 | 2.83 |


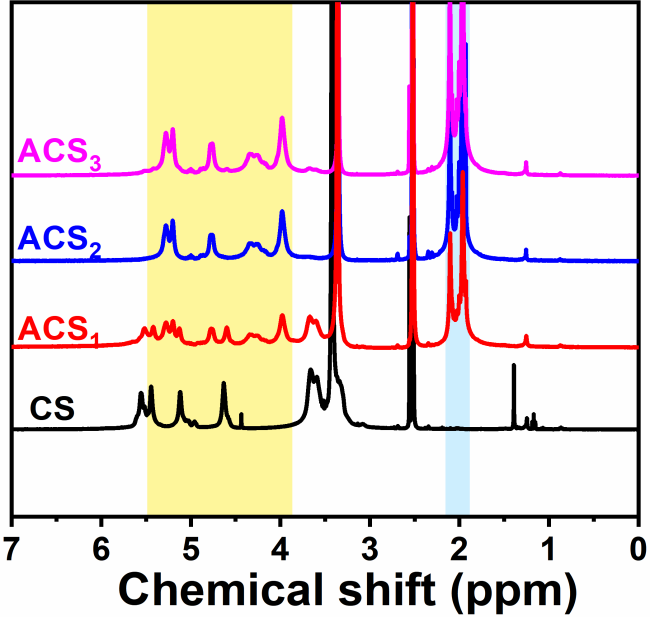


**Fig. S1.** 1H-NMR spectra of CS, ACS_1_, ACS_2_, and ACS_3_.


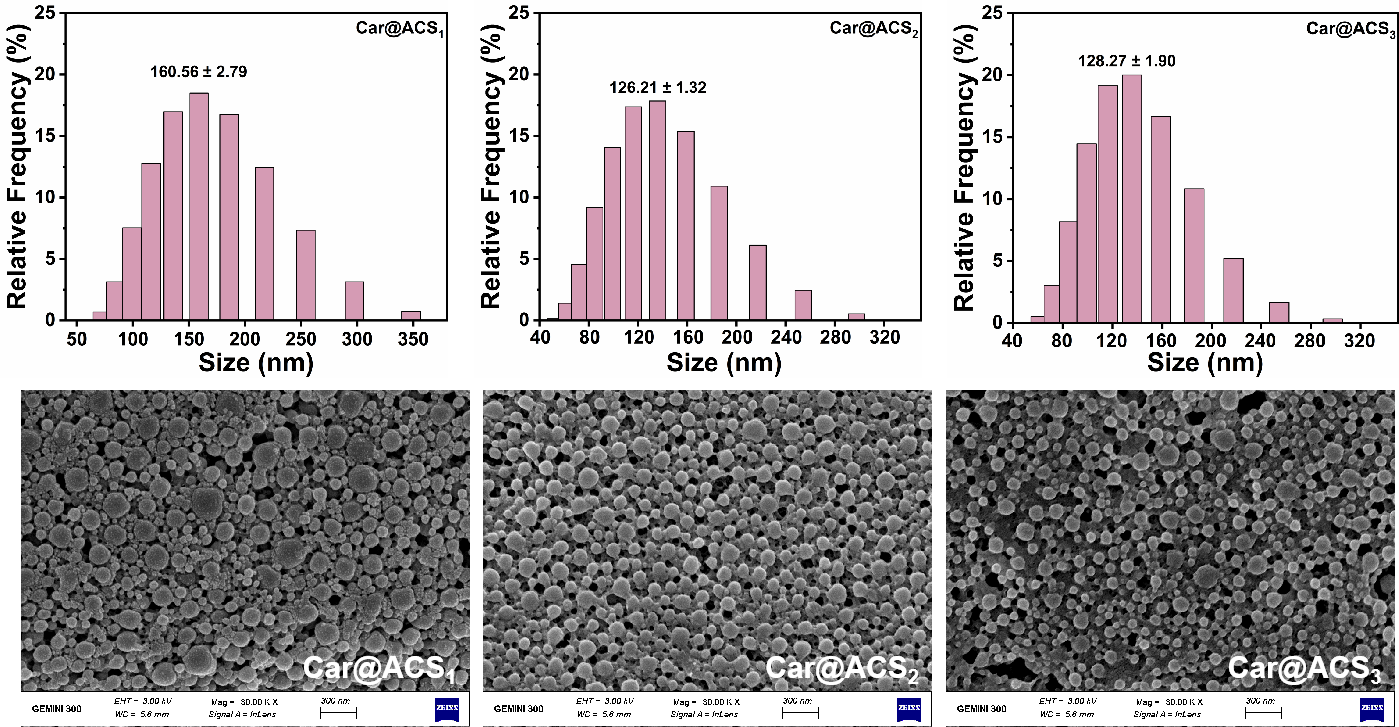


**Fig. S2.** SEM image, size distribution and DLS of different Car@ACS nanoparticles. Zeta potential of different Car@ACS nanoparticle and Car SL.


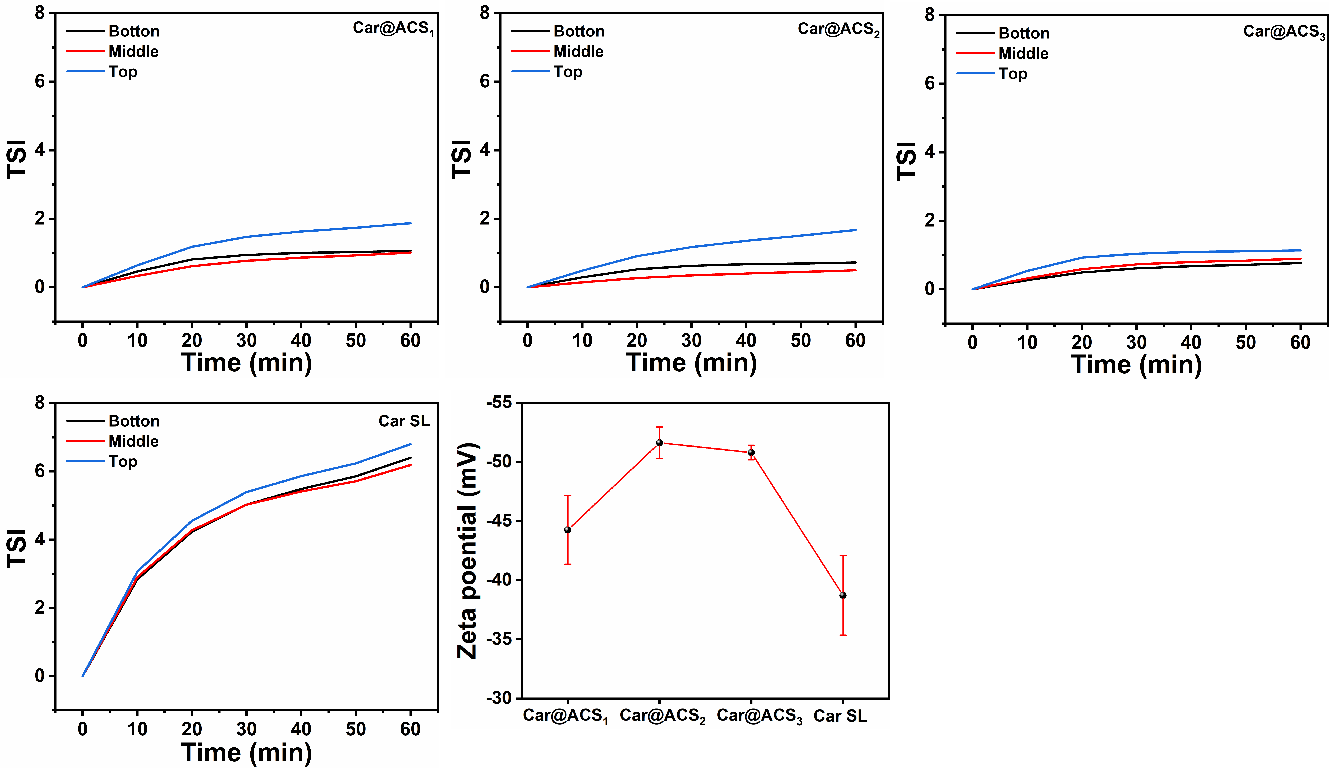


**Fig. S3.** Turbiscan stability index (TSI) values and Zeta potential of Car@ACS_1_, Car@ACS_2_, Car@ACS_3_ and Car SL. A higher TSI value indicated a less stable formulation sample in different part. (Top: 40 – 30 cm, middle: 25 – 15 cm, and bottom: 10 – 0 cm)





**Fig. S4.** The HPLC calibration curve for carvacrol.


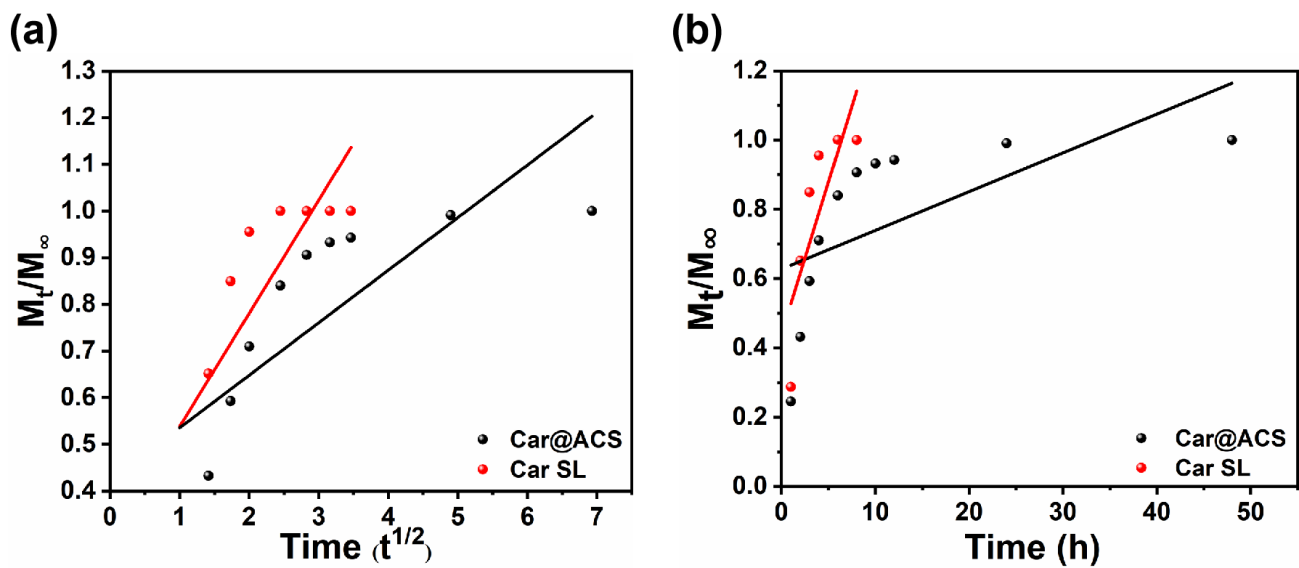


**Fig. S5.** Fitting curves of (a) zero-order model and (b) Higuchi model.

**Table S2.** Release kinetics equations for Car SL and Car@ACS_2_

| **Sample** | **Kinetic model** | **Regression equation** | **R^2^** |
| --- | --- | --- | --- |
| **Car SL** | Zero-order | *y* = 137*x* + 0.326 | 0.784 |
|  | First-order | *y* = –1.080*x* + 0.929 | 0.995 |
|  | Higuchi | *y* = 0.505*x* - 0.104 | 0.884 |
|  | Ritger Peppas | *y* = 43.043*x*^0.531^ | 0.875 |
|  | WeiBull | y = 0.958x - 1.073 | 0.977 |
| **Car@ACS_2_** | Zero-order | *y* = 0.022*x* + 0.410 | 0.569 |
|  | First-order | *y* = -0.054*x* - 0.543 | 0.689 |
|  | Higuchi | *y* = 0.150*x* + 0.196 | 0.768 |
|  | Ritger Peppas | *y* = 27.701*x*^0.432^ | 0.926 |
|  | WeiBull | y = -1.670x - 1.259 | 0.909 |


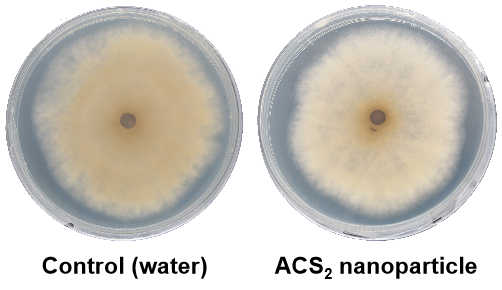


**Fig. S6.** Photographs of control (water) and ACS_5_ nanoparticles (20 μg/mL) against *Botrytis cinerea*.

**Table S3.** The antimicrobial activities of Car Techand Car@ACS_2_ against *Botrytis cinerea*.

| **Samples** | **EC_50_ (μg/mL)** | **Toxicology equations** | **R^2^** | **Confidence interval** |
| --- | --- | --- | --- | --- |
| Car Tech | 26.347 | *y* = 2.445*x - 3.472* | 0.972 | 16.255 - 47.855 |
| Car@ACS_2_ | 32.216 | *y* = 2.405*x - 3.624* | 0.981 | 20.761 - 60.039 |





**Fig. S7.** The Car retention rate of Car SL and Car@ACS_2_ after spraying onto cucumber and peanut leaves.
